# Microbial interspecies interactions: recent findings in syntrophic consortia

**DOI:** 10.3389/fmicb.2015.00477

**Published:** 2015-05-13

**Authors:** Atsushi Kouzuma, Souichiro Kato, Kazuya Watanabe

**Affiliations:** ^1^School of Life Sciences, Tokyo University of Pharmacy and Life SciencesTokyo, Japan; ^2^Bioproduction Research Institute, National Institute of Advanced Industrial Science and TechnologySapporo, Japan

**Keywords:** symbiosis, mutualism, interspecies electron transfer, communication, signal transduction, methanogenesis, microbial fuel cell

## Abstract

Microbes are ubiquitous in our biosphere, and inevitably live in communities. They excrete a variety of metabolites and support the growth of other microbes in a community. According to the law of chemical equilibrium, the consumption of excreted metabolites by recipient microbes can accelerate the metabolism of donor microbes. This is the concept of syntrophy, which is a type of mutualism and governs the metabolism and growth of diverse microbes in natural and engineered ecosystems. A representative example of syntrophy is found in methanogenic communities, where reducing equivalents, e.g., hydrogen and formate, transfer between syntrophic partners. Studies have revealed that microbes involved in syntrophy have evolved molecular mechanisms to establish specific partnerships and interspecies communication, resulting in efficient metabolic cooperation. In addition, recent studies have provided evidence suggesting that microbial interspecies transfer of reducing equivalents also occurs as electric current via biotic (e.g., pili) and abiotic (e.g., conductive mineral and carbon particles) electric conduits. In this review, we describe these findings as examples of sophisticated cooperative behavior between different microbial species. We suggest that these interactions have fundamental roles in shaping the structure and activity of microbial communities.

## Introduction

The pure culture-based techniques developed by Robert Koch and Louis Pasteur have been successfully used for identifying pathogens (Woolhouse and Gaunt, 2007) and obtaining industrially useful microbes (Steele and Stowers, 1991). However, it is generally considered that such methodology has limited capacity for studying the ecology of microbes in natural environments (Amann et al., 1995; Watanabe and Baker, 2000), which markedly differ from pure cultures with respect to nutrient conditions and the presence of interspecies interactions.

Different types of interspecies interactions operate in the biosphere. One of the most common interactions is mutualism, in which two or more different species living in close proximity rely on each other for nutrients, protection, and/or other life functions (Boucher, 1985). A representative case is rhizobial bacteria which fix nitrogen for leguminous plants in return for energy-containing carbohydrates (Long, 1989). Mutualistic relationships are often established between specific partners that are able to sense each other through mechanisms that have evolved to make their interactions more efficient and robust; for instance, plant-produced flavonoids function as signals for initiating legume-rhizobia mutualism (Shaw et al., 2006). Ecologists consider that such specific interactions are more widespread in nature than was previously thought (Doebeli and Knowlton, 1998).

Mutualism also serves as an essential element for shaping microbial communities (Nemergut et al., 2013). Among the different types of mutualistic relationships, syntrophy is a particularly important interspecies interaction that is based on providing trophic benefits for both partners (McInerney et al., 2009). A well-characterized syntrophic interaction occurs between fermentative bacteria (syntrophs) and methanogenic archaea (methanogens), which cooperatively transform organic compounds, such as volatile fatty acids (VFA, including butyrate, propionate, and acetate) into methane (Schink, 1997). This syntrophic interaction is based on the transfer of reducing equivalents, such as hydrogen and formate, between these microbes and is also termed “interspecies electron transfer (IET)” (Schink, 1997). Extensive research has been done to demonstrate the importance of hydrogen/formate-mediated IET in anaerobic digesters (Schink, 1997; McInerney et al., 2009; Stams and Plugge, 2009).

Recently, breakthrough findings, related to interspecies communication (Shimoyama et al., 2009) and direct IET in the form of electric currents (Summers et al., 2010; Kato et al., 2012b), have been reported for microbial interspecies interactions in syntrophic consortia. In the present article, we summarize these findings as examples of the sophisticated microbial interspecies interactions that are fundamental for shaping the composition and structure of microbiota. We do not aim at summarizing current knowledge on IET in methanogenic communities, for which readers are referred to other articles (Sieber et al., 2012; Morris et al., 2013; Shrestha and Rotaru, 2014).

## Interspecies Communication between Specific Partners

Microbes are able to communicate with specific partners for promoting metabolic cooperation. As an example of this phenomenon, this chapter focuses on how interspecies communication is established in a syntrophic consortium and contributes to methanogenesis.

Methane is attracting considerable attention for two different reasons, namely, as a potent greenhouse gas and as a potential source of sustainable energy. Microbial activity is the primary source of methane emission on Earth. Cooperative interactions among microbes belonging to diverse trophic groups, including primary/secondary fermentative bacteria, homoacetogenic bacteria, and hydrogenotrophic/aceticlastic methanogenic archaea; are essential for methanogenesis of organic matter (Schink, 1997). In particular, the close syntrophic interaction that is established between secondary fermentative bacteria (i.e., syntrophs) and hydrogenotrophic methanogens is regarded as the rate-limiting step of methanogenesis, as its stagnation leads to the accumulation of unfavorable metabolites and decay of the entire methanogenic process (Van Lier et al., 1996).

The key reaction for establishing the mutualistic interaction between syntrophs and methanogens is the intercellular transfer of reducing equivalents. For example, syntrophic propionate-oxidizing bacteria acquire energy through the oxidation of propionate into acetate. The reducing equivalents generated through propionate oxidation are used for the reduction of protons to produce H_2_ (Eq. 1).

(1) $$\begin{array}{c} {CH}_{3}{CH}_{2}{COO}^{-}+3H_{2}O\to {CH}_{3}{COO}^{-}+{HCO}_{3}^{-}+H^{+}+3H_{2}\\ \Delta G{}^{\circ}\prime =+76.1kJ\,\;\left(per\,mol\,of\,propionate\right) \end{array}$$

As the Gibbs free energy change of this reaction under the standard condition is positive (endergonic), this reaction is unfavorable as a catabolic reaction and only proceeds when the concentrations of the products (especially H_2_) are maintained at a very low level (McInerney et al., 2008). In methanogenic environments, hydrogenotrophic methanogens efficiently scavenge available hydrogen to produce CH_4_ as a part of their energy metabolism (Eq. 2).

(2) $$\begin{array}{c} {3H}_{2}+3/4{HCO}_{3}^{-}+3/4H^{+}\to 3/4{CH}_{4}+9/4H_{2}O\\ \Delta G{}^{\circ}\prime =-101.7kJ\left(per\,3\,mol\,of\,hydrogen\right) \end{array}$$

As the growth of hydrogenotrophic methanogens is dependent on the supply of H_2_ by hydrogen-producing syntrophs, these two groups of organisms have a mutual nutritional dependence. When the two reactions (Eqs 1 and 2) occur concomitantly, the syntrophic degradation of propionate becomes exergonic (Eq. 3).

(3) $$\begin{array}{c} {CH}_{3}{CH}_{2}{COO}^{-}+3/4H_{2}O\to 3/4{CH}_{4}+{CH}_{3}{COO}^{-}+1/4{HCO}_{3}^{-}+1/4H^{+}\\ \Delta G{}^{\circ}\prime =-25.6kJ\,\;\left(per\,mol\,of\,propionate\right) \end{array}$$

Although syntrophic propionate degradation is sufficient to sustain the growth of syntrophs and hydrogenotrophic methanogens, the Gibbs free energy change of the overall reaction is less than the energy required for the synthesis of ATP from ADP (40–70 kJ per mol of ATP). This feature suggests that syntrophs and methanogens have acquired specific mechanisms for efficient interspecies interaction that enable their survival under such thermodynamically extreme conditions (Jackson and McInerney, 2002).

The transfer of reducing equivalents (i.e., H_2_) from syntrophs to methanogens is regarded as the rate-limiting step of syntrophic methanogenesis (de Bok et al., 2004). The flux of H_2_ between two microbial cells can be estimated based on the Fick’s diffusion law:

(4) $$J=A\cdot D\cdot \frac{C_{p}-C_{c}}{d}$$

In this equation, *J* is the flux of H_2_, *A* is the surface area of the H_2_-producing microbial cells, *D* is the diffusion coefficient of H_2_, *C_p_* and *C_c_* are the concentrations of H_2_ at the surfaces of the H_2_-producing and H_2_-consuming microbial cells, respectively, and *d* is the average distance between H_2_-producing and H_2_-consuming microbial cells. This equation clearly shows that the efficiency of interspecies hydrogen transfer increases with decreasing *d*. Thus, the close proximity or direct physical contact between syntrophs and methanogens is regarded as a critical factor for efficient methanogenesis (Stams, 1994; de Bok et al., 2004; Ishii et al., 2006).

Microbial aggregates, such as granules and biofilms, are frequently observed in methanogenic microbial communities, including those in methanogenic wastewater treatment systems (Skiadas et al., 2003). Fluorescence microscopy has revealed that syntrophs are often found in close proximity to methanogens within such microbial aggregates (Grotenhuis et al., 1991; Imachi et al., 2000). Co-aggregation has also been observed in defined co-cultures of syntrophs and methanogens that do not form aggregates in pure cultures (Ishii et al., 2005). For example, the propionate-oxidizing bacterium *Pelotomaculum thermopropionicum* and hydrogenotrophic methanogen *Methanothermobacter thermautotrophicus* co-aggregate under syntrophic methanogenic conditions (Ishii et al., 2005, 2006). In addition, such studies have shown that the degree of co-aggregation, which is characterized by the abundance and size of aggregates, is influenced by the available growth substrates. Specifically a large number of cell aggregates were observed when the syntrophic cultures were grown on energetically poor substrates, such as propionate, whereas relatively few aggregates were formed in cultures supplemented with energetically rich substrates, such as ethanol. This phenomenon can be explained by the fact that syntrophic methanogenesis from energetically unfavorable substrates requires more efficient interspecies hydrogen transfer than that required for the degradation of energetically favorable substrates, as extremely low hydrogen concentrations are required for syntrophic bacteria to acquire energy through the oxidation of energetically unfavorable substrates.

**FIGURE 1:**
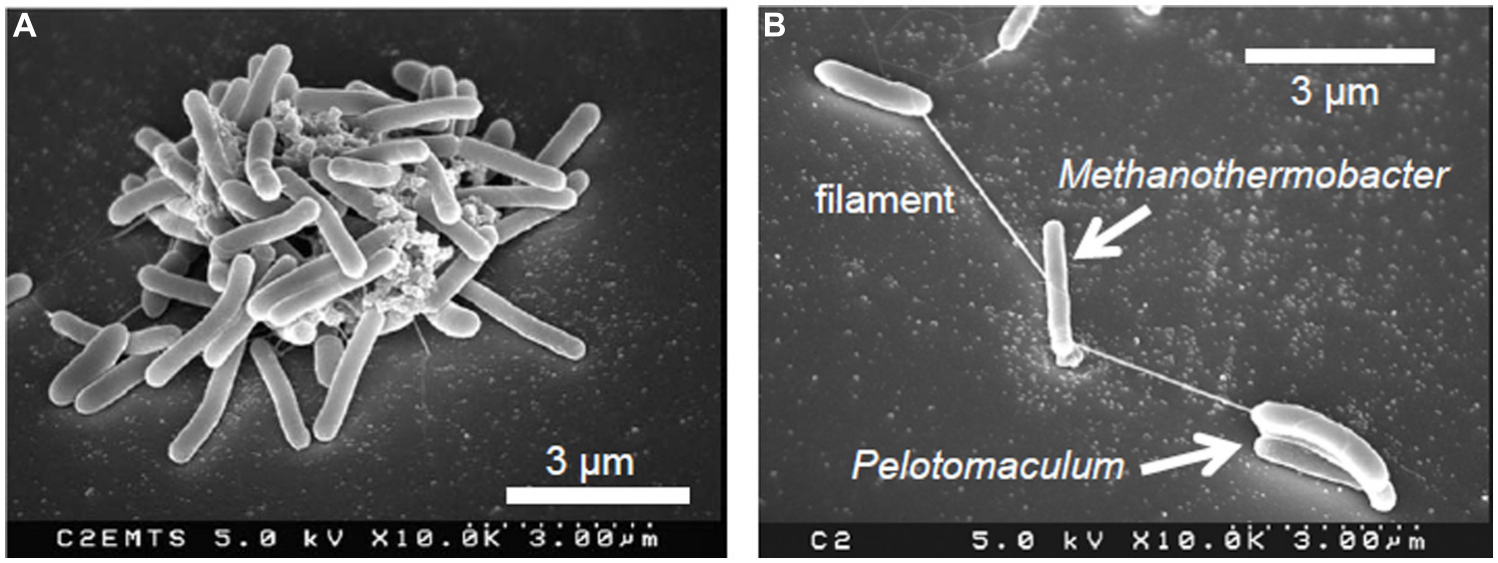
**Scanning electron micrographs of co-cultures of the syntrophic propionate-oxidizing bacterium *Pelotomaculum thermopropionicum* and the hydrogenotrophic methanogen *Methanothermobacter thermautotrophicus* in the middle (A) and early logarithmic growth phase (B)**.

The spatial organization of syntrophs and methanogens is considered to be crucial for efficient methanogenesis. As the close or direct interaction of these microbes is necessary for efficient hydrogen transfer, random cell-to-cell associations with other microbial species may cause the deterioration of methanogenic metabolism. To discriminate their syntrophic partners from other microbial species, syntrophs and methanogens may exploit specific interspecies cell-to-cell recognition systems. This hypothesis has been demonstrated in a syntrophic consortium consisting of *P. thermopropionicum* and *M. thermautotrophicus*, in which conceptually novel interspecies recognition and signaling mechanisms were also found (Shimoyama et al., 2009; Kato and Watanabe, 2010). As stated above, these two microbes produce large co-aggregates that can exceed several 100 mm in diameter during syntrophic methanogenesis from propionate (**Figure 1A**; Ishii et al., 2005). Furthermore, scanning electron microscopy (SEM) has revealed that cells of these two species are often interconnected via filament-like structures in the early- and mid-logarithmic growth phases (**Figure 1B**; Ishii et al., 2005). Database searches using available genomic data of *P. thermopropionicum* (Kosaka et al., 2008) identified putative genes for filamentous appendages in *P. thermopropionicum* (flagella and pili), but not in *M. thermautotrophicus*. The filamentous structures connecting these two microbes were confirmed to be flagella of *P. thermopropionicum* by purification, gel electrophoresis, and N-terminal amino-acid sequencing of the protein derived from the filaments (Shimoyama et al., 2009). Microscopic observation with fluorescently labeled antisera against the major flagellum protein (FliC) of *P. thermopropionicum* further confirmed the origin of these filaments connecting the two species. Although the primary function of flagella is to confer motility, recent studies have demonstrated that flagella have other roles, including adhesion to solid surfaces and environmental sensing (Anderson et al., 2009). As *P. thermopropionicum* is reported to be non-motile (Imachi et al., 2002), it is speculated that *P. thermopropionicum* flagella are specifically utilized for the recognition and/or entrapment of its syntrophic partners (i.e., methanogenic archaea), rather than locomotion.

Certain pathogenic bacteria recognize and adhere to mammalian cells using their flagella (Girón et al., 2002; Guerry, 2007). Among the diverse component proteins that comprise flagella, the major body protein FliC and cap protein FliD have the capacity to attach to mammalian cells (Tasteyre et al., 2001). To examine whether *P. thermopropionicum* FliC and FliD proteins mediate adherence to its syntrophic partner, recombinant FliC and FliD proteins of *P. thermopropionicum* were purified, fluorescently labeled, and mixed with phylogenetically diverse microbial cells (Shimoyama et al., 2009). Flagellar protein-dependent fluorescence was only observed in association with two species of methanogens, *M. thermautotrophicus* and *Methanosaeta thermophila*, which are syntrophic partners of *P. thermopropionicum*. Fluorescent signals were not detected for other microbes, including other methanogens and any of the examined bacterial species, including *P. thermopropionicum* itself. Interestingly, as *M. thermautotrophicus* and *M. thermophila* have major cell-surface components that differ markedly with respect to chemical structures (pseudomurein and glycoprotein, respectively), these results suggest that FliC and FliD of *P. thermopropionicum* adhere to partner organisms via specific cell-surface components, such as cell-surface proteins and/or carbohydrate moieties, rather than major cell-surface structures.

Mammalian immune cells recognize FliC proteins of microbial pathogens using Toll-like receptors and activate intracellular signaling pathways to induce innate immunity (Akira and Takeda, 2004). The existence of such a sensing and signaling network suggests a possibility that the adherence of *P. thermopropionicum* flagellar proteins with the cell-surface components of syntrophically associated methanogens communicates a signal that modifies gene expression and cellular functions in the methanogens. Global transcriptomic analyses of *M. thermautotrophicus* cells demonstrated that the exposure to *P. thermopropionicum* FliD, but not FliC, affects the expression of genes involved in the central metabolism and energy acquisition (Shimoyama et al., 2009). Furthermore, the supplementation of *M. thermautotrophicus* cultures with FliD enhanced methanogenic activity, whereas FliC had no significant effect. Taken together, these findings suggest that the adherence of flagellar cap protein FliD to *M. thermautotrophicus* cells induces the transcription of central catabolic genes, leading to the activation of hydrogenotrophic methanogenesis. As described above, H_2_ in the immediate vicinity of *P. thermopropionicum* cells must be efficiently scavenged for propionate oxidation to proceed. Thus, the specific adherence to its hydrogen-scavenging partner and subsequent activation of hydrogenotrophic methanogenesis is considered to be a sophisticated strategy of propionate-oxidizing syntrophs for survival in competitive microbial communities.

We have described the model that represents one of primary means by which microbes accelerate interspecies hydrogen transfer for syntrophic methanogenesis. In this novel symbiotic relationship, the *P. thermopropionicum* flagellum appears to have two unique functions: it specifically entraps syntrophic partners and mediates specific interspecies signaling. In contrast to well-characterized diffusible chemical signals, such as those involved in quorum sensing, the flagellum-mediated system can only transmit signals to the specific partners. We consider that this specific interspecies signaling is not possible without the high structural diversity of proteins. The molecular mechanism for signaling between *P. thermopropionicum* and *M. thermautotrophicus* is the first example of protein-based interspecies communication between prokaryotes, and we expect that protein-based interspecies communication will also be discovered for diverse microbes in future.

## Direct IET Via Electric Currents

In recent years, researchers have provided evidence suggesting that syntrophy in anaerobic microbiota proceeds not only via the diffusion of electron carriers (e.g., hydrogen and formate), but also via direct IET in the form of electric current between electron-donating and -accepting microbes. A number of dissimilatory iron-reducing bacteria, including *Geobacter metallireducens* (Summers et al., 2010; Rotaru et al., 2014a,b) and *G. sulfurreducens* (Bond and Lovley, 2003; Kato et al., 2012b), have been shown to transfer electrons extracellularly and to generate current in microbial fuel cells (Lovley and Phillips, 1988; Bond and Lovley, 2003). *S*tudies on microbial current generation and extracellular electron transfer (EET) have revealed that outer membrane c-type cytochromes and electrically conductive pilus-like structures, called nanowires, play important roles in these processes in *G. sulfurreducens* (Lovley, 2012). On the other hand, electron-accepting microbes include iron-oxidizing bacteria, such as *Thiobacillus denitrificans* (Kato et al., 2012b), and methanogenic archaea, such as *Methanosaeta harundinacea* (Rotaru et al., 2014b) and *Methanosarcina barkeri* (Rotaru et al., 2014a). *G. sulfurreducens* can accept and use electrons from *G. metallireducens* for fumarate reduction (Summers et al., 2010). Studies have also shown that many of these microbes are capable of accepting electrons from electrodes in bioelectrochemical systems (Gregory et al., 2004; Strycharz et al., 2011; Kato et al., 2012b; Lohner et al., 2014); however, the underlying molecular mechanisms by which these microbes accept electrons from their syntrophic partners and electrodes are less clear than those involved in EET by electron-donating microbes.

The first evidence supporting IET via electric currents was provided by a study using co-cultures of *G. metallireducens* and *G. sulfurreducens* grown in medium containing ethanol as the electron donor and fumarate as the electron acceptor (Summers et al., 2010). In pure culture, *G. metallireducens* is able to metabolize ethanol, but is unable to utilize fumarate as an electron acceptor, whereas the opposite is true for *G. sulfurreducens*. In co-culture, however, these bacteria cooperatively oxidize ethanol with the concomitant reduction of fumarate, and form spherical cell aggregates that are electrically conductive. Fluorescence *in situ* hybridization has revealed that the two *Geobacter* species are closely associated with each other within the aggregates. Notably, cell growth and ethanol consumption proceeded even when co-cultures were inoculated with a *G. sulfurreducens* strain that was unable to utilize hydrogen and formate due to deletion of the genes encoding formate dehydrogenase (*fdnG*) and uptake hydrogenase (*hybL*), demonstrating that syntrophic ethanol metabolism by *G. metallireducens* and *G. sulfurreducens* can occur without interspecies transfer of hydrogen and/or formate. In addition, it was found that a mutation that enhances the production of OmcS, a pili-associated c-type cytochrome that promotes EET to insoluble Fe(III) oxides and electrodes, was selectively introduced into the genome of *G. sulfurreducens* under co-culture conditions, resulting in the acceleration of aggregate formation. In contrast, deletion of the gene encoding OmcS or the structural pilin protein PilA impaired cell growth in the co-cultures. These findings suggest that IET between these *Geobacter* species in cell aggregates occurs via electrical networks comprised of conductive pili and extracellular cytochromes.

Studies have also indicated that IET via direct cell contact is required for syntrophic methanogenesis by *Geobacter* and *Methanosaeta* species (Morita et al., 2011; Rotaru et al., 2014b). Morita et al. (2011) found that microbial aggregates derived from an anaerobic digester converting beer-brewery waste to methane were electrically conductive and exhibited a temperature-dependent response similar to that of conductive biofilms and purified pili preparations of *Geobacter* species. Microbial community analysis of the aggregates using 16S rRNA gene clone library sequencing revealed that *Geobacter* and *Methanosaeta* species were the most abundant bacteria and archaea, respectively, suggesting that these microbes play important roles in syntrophic methanogenesis coupled to ethanol oxidation in the brewery digester. Recently, Rotaru et al. (2014b) have revealed that ethanol is stoichiometrically converted to methane in defined co-cultures of *G. metallireducens* and *M. harundinacea* in accordance with the equation: 2CH_3_CH_2_OH → 3CH_4_ + CO_2_. The complete conversion of ethanol to methane indicates that methanogenesis by *M. harudinacea* occurs not only via the conversion of the acetate produced from ethanol by *G. metallireducens*, but also via the reduction of carbon dioxide by the electrons produced from ethanol oxidation (8H^+^ + 8e^-^ + CO_2_ → CH_4_ + 2H_2_O). However, it is unlikely that the reduction of carbon dioxide occurs via interspecies hydrogen transfer, as *G. metallireducens* does not produce hydrogen during ethanol metabolism, and because *M. harudinacea* cannot utilize hydrogen as an electron donor for the reduction of carbon dioxide. Consistent with this speculation, metatranscriptomic analysis of cells in the co-culture and digester aggregates revealed that the genes for the putative carbon dioxide reduction pathway in *M. harudinacea* and the gene encoding PilA, the structural protein for electrically conductive pili in *Geobacter* species, were highly expressed. Taken together, these results suggest that *M. harudinacea* is capable of directly accepting electrons from *G. metallireducens* for the reduction of carbon dioxide to methane. A recent study by Rotaru et al. (2014a) reported that *M. barkeri* is also able to accept electrons from *G. metallireducens* through the formation of cell aggregates. In addition, the study also revealed that when *M. barkeri* was grown in co-cultures with hydrogen-producing *Pelobacter carbinolicus*, *M. barkeri* utilized hydrogen as an electron donor for carbon dioxide reduction, but did not aggregate with *P. carbinolicus*. These observations demonstrate that close physical contact is needed for direct IET via electric current but not for interspecies hydrogen transfer.

**FIGURE 2:**
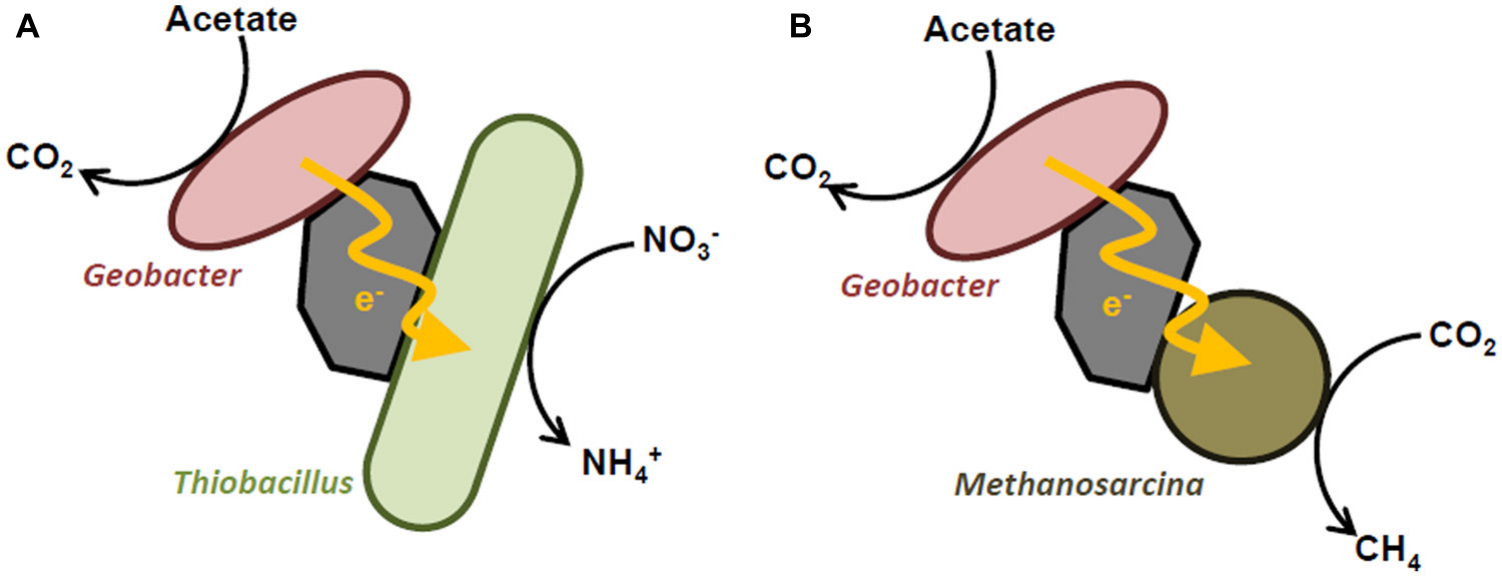
**Schematic diagrams showing electric syntrophy between *Geobacter* and *Thiobacillus denitrificans* (A) and *Methanosarcina* (B)**.

Syntrophic cooperation via IET is also facilitated by electrically conductive substances, including mineral particles and carbon materials (Kato et al., 2012a,b; Liu et al., 2012; Aulenta et al., 2013; Chen et al., 2014a,b; Cruz Viggi et al., 2014). The first experimental evidence for IET mediated by conductive materials was provided by Kato et al. (2012b), who demonstrate that magnetite nanoparticles facilitate IET from *G. sulfurreducens* to *T. denitrificans*, thereby promoting the oxidation of acetate coupled to nitrate reduction (**Figure 2A**). Although syntrophic acetate catabolism by these two bacterial species also occurred in the presence of Fe ions, which functioned as diffusive redox species, the electron transfer rate in the presence of magnetite nanoparticles was more than 10-fold higher than that for the Fe ion-supplemented co-cultures. This finding suggests that conductive magnetite particles serve as electron conduits between *G. sulfurreducens* and *T. denitrificans*, and therefore facilitate direct IET and syntrophic acetate catabolism. In that experiment, the possibility of interspecies hydrogen or formate transfer was excluded by the fact that *T. denitrificans* cannot use these compounds as electron donors. Syntrophic acetate catabolism did not proceed in the absence of iron-oxide nanoparticles and Fe ions, indicating that IET via direct cell contact and conductive biological appendages, such as extracellular cytochromes and pili, did not occur in co-cultures of *G. sulfurreducens* and *T. denitrificans*.

Recent studies have demonstrated that syntrophic methanogenesis is also promoted by the presence of (semi)conductive mineral particles (Kato et al., 2012a; Cruz Viggi et al., 2014). For example, Kato et al. (2012a) found that when magnetite or hematite nanoparticles were added to mixed cultures containing acetate or ethanol as a substrate and rice paddy field soil as inoculum, methanogenesis was significantly accelerated with respect to lag time and production rate. Community analyses based on 16S rRNA genes revealed that *Geobacter* and *Methanosarcina* species were predominant in the enrichment cultures supplemented with magnetite or hematite nanoparticles, suggesting that these (semi)conductive minerals facilitated IET between these microbes, thereby promoting their growth and syntrophic methanogenesis (**Figure 2B**). This notion was further supported by the observation that addition of a specific inhibitor of methanogenesis to the enrichment cultures suppressed not only the growth of the methanogens, but also that of *Geobacter* sp. Cruz Viggi et al. (2014) also reported that the supplementation of methanogenic sludge with magnetite particles enhanced the methane-production rate from propionate, which is a key intermediate in the anaerobic digestion of organic matter, by up to 33%. Based on theoretical calculations, the authors have proposed that IET via electric currents through the magnetite particles is an intrinsically faster electron transfer mechanism compared to interspecies hydrogen transfer. It was also found that methanogenesis in the presence of magnetite particles was less sensitive to external hydrogen partial pressure than that in non-supplemented controls, further supporting the presence of an electron transfer mechanism other than interspecies hydrogen transfer (i.e., IET via electric currents). Given that (semi)conductive minerals are ubiquitously and abundantly present in nature, the findings presented in these reports suggest the possibility that (semi)conductive minerals substantially contribute to microbial catabolic processes, including methanogenesis, in the natural environment by serving as electron conduits.

Conductive carbon materials, including graphite particles (Kato et al., 2012b), granular activated carbon (GAC; Liu et al., 2012; Rotaru et al., 2014b), biochar (Chen et al., 2014b), and carbon cloth (Chen et al., 2014a), are capable of facilitating IET. Although certain carbon materials, such as GAC (Liu et al., 2012) and carbon cloths (Sasaki et al., 2010), are used for enhancing and stabilizing methanogenesis from wastes in anaerobic digesters, it is not fully understood how these materials stimulate methanogenesis. Liu et al. (2012) have reported that the stimulatory effect of GAC on methanogenesis is likely attributable to the high electrical conductivity of this material, which allows electrical connections to be formed between syntrophs and methanogens. In that study, the authors found that the addition of GAC to the co-cultures of *G. metallireducens* and *G. sulfurreducens* markedly accelerated syntrophic ethanol metabolism coupled to fumarate reduction, suggesting that GAC served as an electron conduit, thereby facilitating IET via electric current. A similar stimulatory effect on IET was also observed when *M. barkeri* was grown in co-culture with *G. metallireducens*. In the presence of GAC, co-cultures of *G. metallireducens* and an *omcS*-deleted strain of *G. sulfurreducens* were able to metabolize ethanol with the concomitant reduction of fumarate. It was also found that in GAC-supplemented co-cultures of *G. metallireducens* and *G. sulfurreducens* or *M. barkeri*, cells were tightly associated with GAC, but were not in close contact with each other. These findings indicate that GAC can substitute biological conductive networks that connect electron-donating and -accepting microbes.

Although the above-described studies indicate that certain methanogens, including *Methanosaeta* and *Methanosarcina* species, can accept electrons from their syntrophic partners via direct cell contact and conductive materials, the molecular mechanisms mediating electron uptake by these methanogens are largely unknown. As members of the genera *Methanosaeta* and *Methanosarcina* have membrane-bound cytochromes (Thauer et al., 2008), it is possible that such cell surface-associated conductive proteins may be involved in extracellular electron uptake. Lohner et al. (2014) have provided genetic evidence that *Methanococcus maripaludis* can use electrons accepted from cathode electrodes and use them for the reduction of carbon dioxide to methane in a hydrogenase-independent manner. Methanogenesis from cathodic electrons has also been observed in several undefined enrichment cultures (Cheng et al., 2009; Villano et al., 2010; Villano et al., 2011), suggesting that mechanisms for extracellular electron uptake might be widespread in methanogens. Further studies of genetically accessible methanogens, such as *M. maripaludis* (Moore and Leigh, 2005) and *M. barkeri* (Kohler and Metcalf, 2012), are needed to elucidate the molecular mechanisms underlying methanogenesis involving IET via electric currents.

The finding that IET proceeds via electrical current indicates that microbes can share available energy during anaerobic catabolism in a more direct and efficient manner than previously thought, as electric current-mediated IET allows electrons to be transferred between syntrophic partners at higher rates than interspecies hydrogen/formate transfer (Kato et al., 2012b). Multiple lines of evidence suggest that although conductive pili and extracellular cytochromes play key roles in mediating IET, conductive materials, including mineral and carbon particles, can substitute these biological conductive appendages in IET processes. Given that the synthesis of such biological conduits (e.g., multi-heme cytochromes) requires a large energy investment, it is reasonable to speculate that anaerobic microbes preferentially utilize natural conductive minerals, such as magnetite, as electron conduits for IET. This notion is supported by the observation that the supplementation of culture medium with hematite or magnetite particles suppressed the formation of conductive biofilms during current generation by *G. sulfurreducens* (Kato et al., 2013). These findings indicate that the construction of efficient electrically conductive networks in microbial communities can greatly facilitate methanogenesis and other useful biological processes. A deeper understanding of the complex microbial interactions involving IET will not only help elucidate syntrophic microbial behavior under energy-limited conditions, but will also provide novel strategies for the development of more efficient bioenergy processes.

## Conclusion

Microbes never live alone in natural ecosystems, where their cells are physically and trophically interacting with each other. This article overviewed two fascinating strategies, flagellum-mediated communication and direct IET, which microbes have evolved to facilitate interspecies interactions. Although numerous studies have been done on microbial interspecies interactions, particularly on syntrophy in methanogenic communities, these examples suggest a possibility that there still exist as-yet-unidentified mechanisms that increase the efficiency and robustness of microbial interspecies interactions. In addition, these findings provide us with new opportunities to engineer microbial processes used for environmental protection and bioenergy production. Given the great diversity of microbes in nature, it is likely that there exist a huge repertoire of unique molecular mechanisms for facilitating interspecies interactions. We expect that microbial interspecies interactions will begin to be more prominent in microbiological studies and the potential extent of their influence on microbial ecology, physiology, and evolution is tremendous.

## Conflict of Interest Statement

The authors declare that the research was conducted in the absence of any commercial or financial relationships that could be construed as a potential conflict of interest.

## References

[B1] Akira S. Takeda K. (2004). Toll-like receptor signalling. *Nat. Rev. Immunol.* 4 499–511 10.1038/nri1391 15229469

[B2] Amann R. I. Ludwig W. Schleifer K. H. (1995). Phylogenetic identification and in situ detection of individual microbial cells without cultivation. *Microbiol. Rev.* 59 143–169. 7535888 10.1128/mr.59.1.143-169.1995 PMC239358

[B3] Anderson J. K. Smith T. G. Hoover T. R. (2009). Sense and sensibility: flagellum-mediated gene regulation. *Trends Microbiol.* 18 30–37 10.1016/j.tim.2009.11.001 19942438 PMC2818477

[B4] Aulenta F. Rossetti S. Amalfitano S. Majone M. Tandoi V. (2013). Conductive magnetite nanoparticles accelerate the microbial reductive dechlorination of trichloroethene by promoting interspecies electron transfer processes. *ChemSusChem* 6 433–436 10.1002/cssc.201200748 23401476

[B5] Bond D. R. Lovley D. R. (2003). Electricity production by *Geobacter sulfurreducens* attached to electrodes. *Appl. Environ. Microbiol.* 69 1548–1555 10.1128/AEM.69.3.1548-1555.2003 12620842 PMC150094

[B6] Boucher D. H. (ed). (1985). *The Biology of Mutualism: Ecology and Evolution*. New York, NY: Oxford Univ. Press.

[B7] Chen S. Rotaru A.-E. Liu F. Philips J. Woodard T. L. Nevin K. P. (2014a). Carbon cloth stimulates direct interspecies electron transfer in syntrophic co-cultures. *Bioresour. Technol.* 173 82–86 10.1016/j.biortech.2014.09.009 25285763

[B8] Chen S. Rotaru A.-E. Shrestha P. M. Malvankar N. S. Liu F. Fan W. (2014b). Promoting interspecies electron transfer with biochar. *Sci. Rep.* 4:5019 10.1038/srep05019 PMC4028902 24846283

[B9] Cheng S. Xing D. Call D. F. Logan B. E. (2009). Direct biological conversion of electrical current into methane by electromethanogenesis. *Environ. Sci. Technol.* 43 3953–3958 10.1021/es803531g 19544913

[B10] Cruz Viggi C. Rossetti S. Fazi S. Paiano P. Majone M. Aulenta F. (2014). Magnetite particles triggering a faster and more robust syntrophic pathway of methanogenic propionate degradation. *Environ. Sci. Technol.* 48 7536–7543 10.1021/es5016789 24901501

[B11] de Bok F. A. Plugge C. M. Stams A. J. (2004). Interspecies electron transfer in methanogenic propionate degrading consortia. *Water Res.* 38 1368–1375 10.1016/j.watres.2003.11.028 15016514

[B12] Doebeli M. Knowlton N. (1998). The evolution of interspecific mutualisms. *Proc. Natl. Acad. Sci. U.S.A.* 95 8676–8680 10.1073/pnas.95.15.8676 9671737 PMC21135

[B13] Girón J. A. Torres A. G. Freer E. Kaper J. B. (2002). The flagella of enteropathogenic *Escherichia coli* mediate adherence to epithelial cells. *Mol. Microbiol.* 44 361–379 10.1046/j.1365-2958.2002.02899.x 11972776

[B14] Gregory K. B. Bond D. R. Lovley D. R. (2004). Graphite electrodes as electron donors for anaerobic respiration. *Environ. Microbiol.* 6 596–604 10.1111/j.1462-2920.2004.00593.x 15142248

[B15] Grotenhuis J. T. Smit M. Plugge C. M. Xu Y. S. van Lammeren A. A. Stams A. J. (1991). Bacteriological composition and structure of granular sludge adapted to different substrates. *Appl. Environ. Microbiol.* 57 1942–1949. 1892385 10.1128/aem.57.7.1942-1949.1991 PMC183503

[B16] Guerry P. (2007). Campylobacter flagella: not just for motility. *Trends Microbiol.* 15 456–461 10.1016/j.tim.2007.09.006 17920274

[B17] Imachi H. Sekiguchi Y. Kamagata Y. Hanada S. Ohashi A. Harada H. (2002). *Pelotomaculum thermopropionicum* gen. nov., sp. nov., an anaerobic, thermophilic, syntrophic propionate-oxidizing bacterium. *Int. J. Syst. Evol. Microbiol.* 52 1729–1735 10.1099/ijs.0.02212-0 12361280

[B18] Imachi H. Sekiguchi Y. Kamagata Y. Ohashi A. Harada H. (2000). Cultivation and in situ detection of a thermophilic bacterium capable of oxidizing propionate in syntrophic association with hydrogenotrophic methanogens in a thermophilic methanogenic granular sludge. *Appl. Environ. Microbiol.* 66 3608–3615 10.1128/AEM.66.8.3608-3615.2000 10919827 PMC92191

[B19] Ishii S. Kosaka T. Hori K. Hotta Y. Watanabe K. (2005). Coaggregation facilitates interspecies hydrogen transfer between *Pelotomaculum thermopropionicum* and *Methanothermobacter thermautotrophicus*. *Appl. Environ. Microbiol.* 71 7838–7845 10.1128/AEM.71.12.7838-7845.2005 16332758 PMC1317437

[B20] Ishii S. Kosaka T. Hotta Y. Watanabe K. (2006). Simulating the contribution of coaggregation to interspecies hydrogen flux in syntrophic methanogenic consortia. *Appl. Environ. Microbiol.* 72 5093–5096 10.1128/AEM.00333-06 16820513 PMC1489340

[B21] Jackson B. E. McInerney M. J. (2002). Anaerobic microbial metabolism can proceed close to thermodynamic limits. *Nature* 415 454–456 10.1038/415454a 11807560

[B22] Kato S. Hashimoto K. Watanabe K. (2012a). Methanogenesis facilitated by electric syntrophy via (semi)conductive iron-oxide minerals. *Environ. Microbiol.* 14 1646–1654 10.1111/j.1462-2920.2011.02611.x 22004041

[B23] Kato S. Hashimoto K. Watanabe K. (2012b). Microbial interspecies electron transfer via electric currents through conductive minerals. *Proc. Natl. Acad. Sci. U.S.A.* 109 10042–10046 10.1073/pnas.1117592109 22665802 PMC3382511

[B24] Kato S. Hashimoto K. Watanabe K. (2013). Iron-oxide minerals affect extracellular electron-transfer paths of *Geobacter* spp. *Microbes. Environ.* 28 141–148 10.1264/jsme2.ME12161 23363619 PMC4070692

[B25] Kato S. Watanabe K. (2010). Ecological and evolutionary interactions in syntrophic methanogenic consortia. *Microbes. Environ.* 25 145–151 10.1264/jsme2.ME10122 21576866

[B26] Kohler P. R. A. Metcalf W. W. (2012). Genetic manipulation of *Methanosarcina* spp. *Front. Microbiol.* 3:259 10.3389/fmicb.2012.00259 PMC3403347 22837755

[B27] Kosaka T. Kato S. Shimoyama T. Ishii S. Abe T. Watanabe K. (2008). The genome of *Pelotomaculum thermopropionicum* reveals niche-associated evolution in anaerobic microbiota. *Genome Res.* 18 442–448 10.1101/gr.7136508 18218977 PMC2259108

[B28] Liu F. Rotaru A.-E. Shrestha P. M. Malvankar N. S. Nevin K. P. Lovley D. R. (2012). Promoting direct interspecies electron transfer with activated carbon. *Energy Environ. Sci.* 5 8982–8989 10.1039/c2ee22459c

[B29] Lohner S. T. Deutzmann J. S. Logan B. E. Leigh J. Spormann A. M. (2014). Hydrogenase-independent uptake and metabolism of electrons by the archaeon *Methanococcus maripaludis*. *ISME J.* 8 1673–1681 10.1038/ismej.2014.82 24844759 PMC4817615

[B30] Long S. R. (1989). Rhizobium-legume nodulation: life together in the underground. *Cell* 56 203–214 10.1016/0092-8674(89)90893-3 2643474

[B31] Lovley D. R. (2012). Electromicrobiology. *Annu. Rev. Microbiol.* 66 391–409 10.1146/annurev-micro-092611-150104 22746334

[B32] Lovley D. R. Phillips E. J. (1988). Novel mode of microbial energy metabolism: organic carbon oxidation coupled to dissimilatory reduction of iron or manganese. *Appl. Environ. Microbiol.* 54 1472–1480. 16347658 10.1128/aem.54.6.1472-1480.1988 PMC202682

[B33] McInerney M. J. Sieber J. R. Gunsalus R. P. (2009). Syntrophy in anaerobic global carbon cycles. *Cur. Opin. Biotechnol.* 20 623–632 10.1016/j.copbio.2009.10.001 PMC2790021 19897353

[B34] McInerney M. J. Struchtemeyer C. G. Sieber J. Mouttaki H. Stams A. J. Schink B. (2008). Physiology, ecology, phylogeny, and genomics of microorganisms capable of syntrophic metabolism. *Ann. N. Y. Acad. Sci.* 1125 58–72 10.1196/annals.1419.005 18378587

[B35] Moore B. C. Leigh J. A. (2005). Markerless mutagenesis in *Methanococcus maripaludis* demonstrates roles for alanine dehydrogenase, alanine racemase, and alanine permease. *J. Bacteriol.* 187 972–979 10.1128/JB.187.3.972-979.2005 15659675 PMC545699

[B36] Morita M. Malvankar N. S. Franks A. E. Summers Z. M. Giloteaux L. Rotaru A.-E. (2011). Potential for direct interspecies electron transfer in methanogenic wastewater digester aggregates. *MBio* 2:e00159-11. 10.1128/mBio.00159-11 PMC3157894 21862629

[B37] Morris B. E. Henneberger R. Huber H. Moissl-Eichinger C. (2013). Microbial syntrophy: interaction for the common good. *FEMS Microbiol. Rev.* 37 384–406 10.1111/1574-6976.12019 23480449

[B38] Nemergut D. R. Schmidt S. K. Fukami T. O’Neill S. P. Bilinski T. M. Stanish L. F. (2013). Patterns and processes of microbial community assembly. *Microbiol. Mol. Biol. Rev.* 77 342–356 10.1128/MMBR.00051-12 24006468 PMC3811611

[B39] Rotaru A.-E. Shrestha P. M. Liu F. Markovaite B. Chen S. Nevin K. (2014a). Direct interspecies electron transfer between *Geobacter metallireducens* and *Methanosarcina barkeri*. *Appl. Environ. Microbiol.* 80 4599–4605 10.1128/AEM.00895-14 24837373 PMC4148795

[B40] Rotaru A.-E. Shrestha P. M. Liu F. Shrestha M. Shrestha D. Embree M. (2014b). A new model for electron flow during anaerobic digestion: direct interspecies electron transfer to *Methanosaeta* for the reduction of carbon dioxide to methane. *Energy Environ. Sci.* 7 408–415 10.1039/C3EE42189A

[B41] Sasaki K. Morita M. Hirano S.-I. Sasaki D. Ohmura N. Igarashi Y. (2010). Efficient degradation of rice straw in the reactors packed by carbon fiber textiles. *Appl. Microbiol. Biotechnol.* 87 1579–1586 10.1007/s00253-010-2667-3 20512325

[B42] Schink B. (1997). Energetics of syntrophic cooperation in methanogenic degradation. *Microbiol. Mol. Biol. Rev.* 61 262–280. 9184013 10.1128/mmbr.61.2.262-280.1997 PMC232610

[B43] Shaw L. J. Morris P. Hooker J. E. (2006). Perception and modification of plant flavonoid signals by rhizosphere microorganisms. *Environ. Microbiol.* 8 1867–1880 10.1111/j.1462-2920.2006.01141.x 17014487

[B44] Shimoyama T. Kato S. Ishii S. Watanabe K. (2009). Flagellum mediates symbiosis. *Science* 323:1574 10.1126/science.1170086 19299611

[B45] Shrestha P. M. Rotaru A. E. (2014). Plugging in or going wireless: strategies for interspecies electron transfer. *Front. Microbiol.* 5:237 10.3389/fmicb.2014.00237 PMC4032928 24904551

[B46] Sieber J. R. McInerney M. J. Gunsalus R. P. (2012). Genomic insights into syntrophy: the paradigm for anaerobic metabolic cooperation. *Ann. Rev. Microbiol.* 66 429–452 10.1146/annurev-micro-090110-102844 22803797

[B47] Skiadas I. V. Gavala H. N. Schmidt J. E. Ahring B. K. (2003). Anaerobic granular sludge and biofilm reactors. *Adv. Biochem. Eng. Biotechnol.* 82 35–67 10.1007/3-540-45838-7_2 12747565

[B48] Stams A. J. (1994). Metabolic interactions between anaerobic bacteria in methanogenic environments. *Antonie Van Leeuwenhoek* 66 271–294 10.1007/BF00871644 7747937

[B49] Stams A. J. M. Plugge C. M. (2009). Electron transfer in syntrophic communities of anaerobic bacteria and archaea. *Nat. Rev. Microbiol.* 7 568–577 10.1038/nrmicro2166 19609258

[B50] Steele D. B. Stowers M. D. (1991). Techniques for selection of industrially important microorganisms. *Annu. Rev. Microbiol.* 45 89–106 10.1146/annurev.mi.45.100191.000513 1741626

[B51] Strycharz S. M. Glaven R. H. Coppi M. V Gannon S. M. Perpetua L. A Liu A. (2011). Gene expression and deletion analysis of mechanisms for electron transfer from electrodes to *Geobacter sulfurreducens*. *Bioelectrochemistry* 80 142–150 10.1016/j.bioelechem.2010.07.005 20696622

[B52] Summers Z. M. Fogarty H. E. Leang C. Franks A. E. Malvankar N. S. Lovley D. R. (2010). Direct exchange of electrons within aggregates of an evolved syntrophic coculture of anaerobic bacteria. *Science* 330 1413–1415 10.1126/science.1196526 21127257

[B53] Tasteyre A. Barc M. C. Collignon A. Boureau H. Karjalainen T. (2001). Role of FliC and FliD flagellar proteins of *Clostridium* difficile in adherence and gut colonization. *Infect. Immun.* 69 7937–7940 10.1128/IAI.69.12.7937-7940.2001 11705981 PMC98895

[B54] Thauer R. K. Kaster A.-K. Seedorf H. Buckel W. Hedderich R. (2008). Methanogenic archaea: ecologically relevant differences in energy conservation. *Nat. Rev. Microbiol.* 6 579–591 10.1038/nrmicro1931 18587410

[B55] Van Lier J. B. Martin J. L. S. Lettinga G. (1996). Effect of temperature on the anaerobic thermophilic conversion of volatile fatty acids by dispersed and granular sludge. *Water Res.* 30 199–207 10.1016/0043-1354(95)00107-V

[B56] Villano M. Aulenta F. Ciucci C. Ferri T. Giuliano A. Majone M. (2010). Bioelectrochemical reduction of CO2 to CH4 via direct and indirect extracellular electron transfer by a hydrogenophilic methanogenic culture. *Bioresour. Technol.* 101 3085–3090 10.1016/j.biortech.2009.12.077 20074943

[B57] Villano M. Monaco G. Aulenta F. Majone M. (2011). Electrochemically assisted methane production in a biofilm reactor. *J. Power Sources* 196 9467–9472 10.1016/j.jpowsour.2011.07.016

[B58] Watanabe K. Baker P. W. (2000). Environmentally relevant microorganisms. *J. Biosci. Bioeng.* 89 1–11 10.1016/S1389-1723(00)88043-3 16232691

[B59] Woolhouse M. Gaunt E. (2007). Ecological origins of novel human pathogens. *Crit. Rev. Microbiol*. 33 231–242 10.1080/10408410701647560 18033594

